# A Case of Hydrophobia from the Bite of a Pole-Cat

**Published:** 1859-02

**Authors:** 


### *A Case of Hydrophobia from the Bite of a Pole-Cat—Tracheotomy.* By R. De Jernett, M. D., of Greenville, Texas.

[The following well written report presents, in a graphic manner, the details of one of those touching and painful cases, which too often present themselves as gloomy episodes in the physician's life. The experiment with the operation of Tracheotomy, and the Doctor's inquiries in relation to the nature of the animal poison producing hydrophobia, are subjects of deep interest to the profession, and render the case one of much importance, as a record of that mysterious and terrific affection.]

Called February 24th to see Amanda S——, age ten years. About the 8th of January she was bitten by a pole-cat, in the night, while sleeping.

Her father hearing her cry, went to her relief, and had to choke the cat before he could disengage its hold. He remarked, it was sucking the blood from the wound, which was inflicted on right side of her mouth, in the lip. It was thrown out, and killed by a dog, which was also bitten, but did not manifest any symptoms of the disease up to the 24th February, when he was killed, fearing the disease might yet be developed.

The bite on A. S—— swelled the face, and was painful for four days; then healed as a bite of any kind would, and her health was good until Sunday, Feb. 21st, when her parents observed she was stupid and inclined to sleep. The patient spoke of an itching sensation in the cicatrix.

22d. Patient slept most of the day, and when not sleeping, rather petulant; still complains of itching in the cicatrix; appetite very good.

23d. Symptoms, so far as could be learned, were very much as yesterday, with this exception—has no appetite to eat.

24th. Patient arose from bed before day, and said she felt better than she had for three days; but in the course of an hour complained of spasms about the fauces, in attempting to swallow fluids; yet she could swallow solids with impunity. The family became alarmed, and Dr. Patterson was called in. The Doctor told me, when he was getting the history of the case, that he had some fears it was a case of hydrophobia; but, not being satisfied, and as the case presented some symptoms of worms, he gave a dose of calomel, and left.

The case grew worse very fast, and Dr. P. was sent for again at 3 o'clock P. M. He found her very restless, with jactitation of limbs and occasional slight spasms; he requested the parents to send for me.

When I saw her, 10 o'clock P. M., she was in bed, and very restless. Her father brought her to the fire, and seated her in a large rocking-chair, and she assumed an erect position, with her head rather thrown back, her face flushed; in the countenance was depicted a fearful expression of anxiety. She addressed me in a sobbing tone of voice: said—"I am bitten by a pole-cat—can you cure me?"—then seemed to smile with a forced effort, her face being very much contorted. I was informed by Dr. P. that her pulse had been low and irregular during the evening, the extremities had been cold and moist. Dr. P. had given ammonia, quinine, and some of the anti-spasmodics, with no other effect than that of raising the pulse. I asked her if she had any pain? She replied yes—in the forehead, the back of the neck, and, at times, under the sternum. We gave opium, with a hope of tranquilizing the system and procuring sleep, but in vain. She said there was a mat of long hair in her right eye. I could not see anything, though it was red, and running water—the left was also discharging tears, but not so much as the right; the pupils of both, very much dilated.

11 o'clock P. M. Pulse irregular, but frequent; extremities cold and moist; stomach irritable, constantly spitting a tenacious mucus, and complained of something rising in her throat about the size of her little finger, and on trying to get it out, it would slip down. By pressing forward the tongue with a spoon, I could see a tenacious and frothy substance rise up, in a conical shape, to the posterior nares, which interfered with the free action of the epiglottis; she would become restless, and throw herself back suddenly, as if badly frightened. Her mouth was widely opened during the inspection. When bidden, she would do anything that promised relief. The panic either proceeded from the difficulty in respiration, caused by this substance rising in the throat, or from the slipping of the spoon on the tongue, in her efforts to breathe; the latter is probable, because titillating the skin on any part of the body, or passing a current of air on the skin, produced these shuddering tremors.

I had a cup of water brought, and requested her to take some. She said: "I want it, and will try to drink." Violent agitation of the whole body supervened; finally, rallying sufficient power, she clutched it with both hands, and with a quick movement put it to her mouth. It did not more than touch the mouth, when it was thrown off with violence, and the body convulsed. She was also tried with milk, and with the same result. Her eyes were always directed to one side of the fluid till the moment of seizing it.

12 o'clock. Took about six ounces of blood from the arm, and gave two grains of opium; sinapisms to the extremities; but could not be retained, owing to restlessness.

1 o'clock A. M. Gave, by inhalation, two drachms of chloroform, which only exasperated the symptoms. Her symptoms all grew worse, vomiting came on, and at times delirium, when she would spring from imaginary evils, and halloo at the top of her voice, and occasionally bite the bedclothes.

Standing by, only to witness the futility of the means employed — while the disease was clinging on with unrelenting tenacity — we resolved to act upon the suggestion of Dr. Reynolds of Bellevue Hospital, to open the trachea, and introduce a tube. I proceeded to do so, and she breathed somewhat easier. In this, our *dernier resort*, we did not entertain a sanguine hope of success, for we were expecting death to occur every minute; but asphyxia seemed to be the threatening evil. If this had been done twelve hours sooner, I believe it would have saved the patient; but death occurred suddenly, in a hard convulsion, at 4 o'clock A. M., half hour after the trachea was opened, and sixteen hours from the time the disease was fully developed. The cicatrix, after death, was of a livid hue. No post-mortem examination.

Most writers believe this disease results from the entrance into the blood of the poison of a rabid animal. To this opinion, I cannot be reconciled; for this, with five other cases, within the last six years, have been bitten by these cats, and only two escaped the disease; and in one of them an extensive ulceration was set up in and about the wound. Only one dog has been known to have the disease during these six years. These cats are numerous in this country, and our dogs kill them frequently, and are bitten by the cats, yet the dogs do not have the disease.

I would like that some writer, able to do this subject justice, would give his views on the above facts.—*Southern Med. and Surg. Journal.*

